# The Use of Aromatic Sulphuric Acid in Necrosis

**Published:** 1876-10

**Authors:** Ephraim Cutter

**Affiliations:** Cambridge


### THE USE OF AROMATIC SULPHURIC ACID IN NECROSIS.

BY EPHRAIM CUTTER, M.D., CAMBRIDGE.

April 10th, 1875, Dr. A., of Worcester, requested the writer to remove the necrosed alveolar process of his wife's sister. She was of middle age, pale, thin, weak, anxious and worn. She had suffered much with her teeth. Her upper right middle and two lateral incisors were found to be loose, and their lower edges hanging below the line of their fellows. There was a fungoid, spongy swelling over the front of the diseased process. When this was pressed, pus freely exuded from several openings, and also from a softish, elastic swelling as large as a hazel nut, situated at the dome of the hard palate inside the month. The loosened teeth could be freely moved in every direction with the thumb and fingers. The roots of the teeth distinctly grated against the sound alveolar process. There was a complete separation of the teeth and the bone. Dr. A. said that he had thought of using the aromatic sulphuric acid, but that the disease was so extensive and the separation so complete, that he regarded it as useless to try to save the teeth in any way. It appeared to the consulter, however, while the surgical extirpation would be effective and justifiable, that if free incisions were made into the swollen and spongy gums, there would be an evacuation of the contents of the dilated capillaries and abscesses; that a healthy action would be promoted by relieving this unnatural distention, and that the necrosed bone might be slowly removed by the stimulation of the aromatic sulphuric acid topically applied, without destroying the teeth. It was thought that then the periosteum would lay down new bone in place of the old, and refasten the teeth in their old place. It was agreed to employ the following:

R. Aromatic sulphuric acid..................... dr. j.
Aquæ........................................ oz. j.

By means of a half-ounce syringe supplied with a small ivory tip, one inch and a half long, and one-eighth inch in diameter, the acid solution was injected at first twice a day and afterwards once a day. About two drachms were used at each injection. The syringe tip was deeply buried into the soft tissues through one of the openings. Pus would freely exude from the other openings, even from that in the top of the mouth after each injection.

Tonics were administered. A diet of animal food and unbolted wheat was rigidly maintained.

From the outset of this departure a marked improvement in the soft tissues occurred. But the teeth remained loose and dangling, and Dr. A. thought their recovery doubtful. It was re-suggested that it would be an easy thing to remove them at any time if they did not reset, but that the process of replacing old with new bone was of necessity a slow one.

In about forty days the outer incisor became solidly fixed in its old site. Then the next incisor also tightened. The middle incisor tightened slowly. In November following it could be very slightly moved, but its edge was a little below the line of the other teeth. The other two incisors were as stiff as they ever were. A few spiculæ of bone were removed from the front of the alveolar process during the period of the treatment. In the meantime the general health of the patient improved greatly. She gained in weight, color and strength. At the present time (July, 1876) she is entirely recovered.

We think it is reasonable to connect the result in this case with the means employed, the acid, the tonics, and the food.

Dr. Atkinson, of New York, has reported some remarkable instances of cure of necrosis by this agent, used in its full strength, it is said. It hastens the disintegrating and separating processes, and at the same time destroys the germs of parasitic micographic growths in the dead and dying bone. According to Dr Atkinson, it does not act unhealthily upon sound tissues whose vital connections are unimpaired. No substances stand higher than the mineral acids as antiseptics and destroyers of bacteria, amœbæ, and vegetations of animal secretions. Were it not for their caustic effects they would long ago have supplanted carbolic acid.—*Boston Med. & Surg. Jour.*

